# A cellular mechanism of interactions between pain and depression

**DOI:** 10.1186/1744-8069-10-S1-O10

**Published:** 2014-12-15

**Authors:** Hyangin Kim, Michael F McCabe, Grewo Lim, Lucy Chen, Jianren Mao

**Affiliations:** 1 MGH Center for Translational Pain Research, Department of Anesthesia, Critical Care and Pain Medicine, Massachusetts General Hospital, Harvard Medical School, Boston, MA 02114, USA

## Background

Depression is a common comorbid condition of chronic pain. The current mainstay of managing comorbid chronic pain uses combination drug therapy including opioid analgesics and antidepressants. However, the cellular mechanism underlying the comorbid interaction between chronic pain and depression remains unclear.

## Materials and methods

Using rat models of genetically predisposed [Wistar-Kyoto (WKY) rats] or induced depressive behavior combined with persistent nociception, we examined whether 1) brain IDO1 (a rate-limiting enzyme in tryptophan metabolism) expression and activity would be increased in rats with genetically predisposed or induced depressive behavior, 2) rats with depressive behavior would exhibit exacerbated nociceptive behavior, and 3) brain IDO1 upregulation would be contributory to both depressive and nociceptive behaviors. Depressive behaviors were assessed by using forced swimming test, sucrose preference test, tail suspension test, and open field test. Nociceptive behaviors were assessed using von Frey filaments and hind-paw withdrawal to radiant heat stimulation. RT-PCR, Western blotting, HPLC, ELISA, immunohistochemistry and cell culture were used in the study.

## Results

We demonstrate that brain IDO1 expression critically contributes to the comorbid interaction between pain and depression.(1) IDO1 expression was elevated in the hippocampus of rats with either genetically predisposed or anhedonia-induced depressive behavior. (2) Rats with elevated IDO1 expression exhibited a lower baseline mechanical and thermal nociceptive threshold, whereas depressive behavior was exacerbated in rats with persistent nociception.(3) IDO1 upregulation was mediated by the IL-6/JAK/STAT signaling, resulting in a shift of tryptophan metabolism toward the IDO pathway as well as a low serotonin content in the hippocampus. (4) Inhibition of IDO1 activity or IL-6/JAK/STAT-mediated IDO1 upregulation concurrently attenuated nociceptive and depressive behaviors in the same rats.

## Conclusions

The results indicate that brain IDO1 expression critically contributes to the comorbid interaction between pain and depression and suggest that targeting brain IDO1 activity may offer a new therapeutic method for the treatment of comorbid chronic pain.

